# Whole-Genome Sequencing of Six *Borrelia miyamotoi* Clinical Strains Isolated in Russia

**DOI:** 10.1128/genomeA.01424-17

**Published:** 2018-01-04

**Authors:** Konstantin V. Kuleshov, Joris Koetsveld, Irina A. Goptar, Mikhail L. Markelov, Nadezhda M. Kolyasnikova, Denis S. Sarksyan, Marina G. Toporkova, Nina P. Kirdyashkina, German A. Shipulin, Joppe W. Hovius, Alexander E. Platonov

**Affiliations:** a Central Research Institute of Epidemiology, Moscow, Russia; b Academic Medical Center, University of Amsterdam, Amsterdam, The Netherlands; c Izhevsk State Medical Academy, Izhevsk, Russia; d Medical Association “Novaya Bolnitsa,” Yekaterinburg, Russia; e Kovalenko All-Russian Research Institute for Experimental Veterinary Medicine, Moscow, Russia; f Chumakov Federal Scientific Center for Research and Development of Immunobiological Products of Russian Academy of Sciences, Moscow, Russia; g Research Institute of Occupational Health, Moscow, Russia

## Abstract

Here, we report the whole-genome sequence of six clinical *Borrelia miyamotoi* isolates from the Russian Federation. Using two independent next-generation sequencing platforms, we determined the complete sequence of the chromosome and several plasmids. All strains have an Asian genotype with 99.8% chromosome nucleotide similarity with *B. miyamotoi* strain FR64b.

## GENOME ANNOUNCEMENT

Studies on the emerging human tick-borne pathogen, *Borrelia miyamotoi*, are cumbersome, since only a limited set of strains are available and sequenced to date (1–4). We previously isolated six clinical strains from blood of Russian patients with acute *Borrelia miyamotoi* disease in Izhevsk City (strains Izh-4, Izh-5, Izh-14, and Izh-16) and Yekaterinburg City (strains Yekat-1 and Yekat-6) in 2016 (5).

Low-passage isolates were grown *in vitro* and total DNA was extracted using the DNeasy blood and tissue kit and Qiagen Tip-100 prep (Qiagen) for sequencing using MiSeq and MinION platforms, respectively. In addition, to increase the reliability of the assembly of individual plasmids, we separated DNA by pulsed-field gel electrophoresis (PFGE); 8 to 11 extrachromosomal fragments per isolate—ranging from 5 to 73 kb—were cut out from gels and dissolved in Agarose Dissolving Buffer (Zymoresearch). DNA was extracted using the DNeasy blood and tissue kit, and DNA libraries were prepared using the NexteraXT DNA library kit (Illumina), with a distinct barcode for each fragment. Next, DNA libraries were sequenced using the MiSeq platform and 500-cycle V2 reagent kit (Illumina). Adapter sequences were removed from the Illumina reads by BBTools (https://sourceforge.net/projects/bbmap/). The Native barcoding kit 1D (EXP-NBD103) was used together with the Ligation sequencing kit (SQK-LSK108) to prepare Nanopore sequencing libraries from total DNA. One R9.4 MinION flow cell was used for six multiplexed DNA samples. Base calling of MinION sequences was performed using Albacore v1.1.0, and adapters were removed by Porechop (https://github.com/rrwick/Porechop).

The hybrid *de novo* assembly of Illumina and Nanopore reads was done using Unicycler v0.3.1 (6). We performed separate assemblies of Illumina reads relating to a particular PFGE fragment, aided by the long corresponding Nanopore reads. Subsequently, the contigs from independent assemblies of each isolate were compared and clustered (when >98% similar) by CD-HIT-EST (https://github.com/weizhongli/cdhit) in order to leave only one representative contig from each cluster. The accuracy of our assembly was checked by mapping back short and long reads to each individual contig with strict parameters. Subsequently, contigs containing gaps and/or inconsistent mapping, detected by manual curation and analysis by the REAPR v1.0.18 tool (7), were removed.

We obtained 17 to 20 contigs for each isolate with a read coverage for each contig more than 200×. These contigs included the complete linear chromosomes, several complete plasmids (lp72, cp2, lp41, lp23, and lp6), complete or incomplete hypothetical plasmids (characterized by previously unknown variants of the PFam32 gene [8]), and nontypeable contigs (without the PFam32 gene). Linear chromosomes of the six clinical isolates ranged from 906,129 bp to 906,582 bp and included 828 or 829 protein-coding sequences (CDS), 3 rRNAs, and 31 tRNAs. Interestingly, all isolates had similar lp41 plasmids, yet with highly variable 3′ ends due to different content of genes cassette coding immunodominant variable major proteins (VMP) and including VMP variant expressed by an isolate (4, 9).

Our findings form the basis for future comparative genomics of *B. miyamotoi* isolates and will stimulate further fundamental research, as well as the development of molecular diagnostic tools and epidemiological surveillance of this emerging human pathogen.

### Accession number(s).

The sequences of the chromosomes and complete or incomplete plasmids of Izh-4, Izh-5, Izh-14, Izh-16, Yekat-1, and Yekat-6 isolates were deposited in the GenBank/DDBJ/EMBL database under accession numbers CP024390 to CP024407, CP024205 to CP024222, CP024371 to CP024389, CP024351 to CP024370, CP024333 to CP024350, and CP024316 to CP024332, respectively (BioProject PRJNA406856 and BioSamples SAMN07572561, SAMN07572562, SAMN07572563, SAMN07572564, SAMN07572565, and SAMN07572566).
